# Hypoxia and hypoxia-inducible factors in neuroblastoma

**DOI:** 10.1007/s00441-017-2701-1

**Published:** 2017-10-14

**Authors:** Sven Påhlman, Sofie Mohlin

**Affiliations:** 1 Translational Cancer Research, Department of Laboratory Medicine, Lund University, Medicon Village, Scheelevägen 8, 223 81 Lund, Sweden; 2 Pediatric Oncology & Hematology, Department of Clinical Sciences, Lund University, Lund, Sweden

**Keywords:** Neuroblastoma, Hypoxia, Hypoxia-inducible factor, Vascularization, Cancer stem cell

## Abstract

Hypoxia (i.e., low oxygen levels) is a known feature of aggressive tumors. Cells, including tumor cells, respond to conditions of insufficient oxygen by activating a transcriptional program mainly driven by hypoxia-inducible factors (HIF)-1 and HIF-2. Both HIF-1α and HIF-2α expression levels have been shown to correlate to patient outcome in various tumor forms and in neuroblastoma, a solid childhood tumor of the sympathetic nervous system, in particular, HIF-2α marks a subpopulation of immature neural crest-like perivascularly located cells and associates with aggressive disease and distant metastasis. It has for long been recognized that the HIF-α subunits are oxygen-dependently regulated at the post-translational level, via ubiquitination and proteasomal degradation. Evidence of oxygen-independent mechanisms of regulation, transcriptional control of *EPAS1/HIF2A* and possible cytoplasmic activities of HIF-2α has also emerged during recent years. In this review, we discuss these non-conventional actions of HIF-2α, its putative role as a therapeutic target and the constraints it carries, as well as the importance of HIF-2 activity in a vascularized setting, the so-called pseudo-hypoxic state.

## Oxygenation of solid tumors

It is now well established that solid tumors cannot grow larger than a couple of mm without being vascularized. The degree of vascularization varies between individual tumors and between different tumor forms but, generally, the blood vessels that vascularize tumors are malformed and sub-functional (Semenza 2014). As a result, most parts of solid tumors are sub-optimally oxygenized, which has been demonstrated in numerous studies in which the tumor- and surrounding tissue oxygen tensions have been measured directly with oxygen electrodes (Vaupel et al. 2004). One general conclusion that can be drawn from these observations is that tumor cells also survive and proliferate under low oxygen (hypoxic) conditions, a conclusion solidly confirmed by in vitro studies. One important consequence of solid tumors being poorly oxygenated and adapted to a hypoxic milieu is the hypoxia-induced activation of the transcription and synthesis of pro-angiogenic factors such as VEGF, with subsequent ingrowth of blood vessels and formation of a tumor. Thus, in solid tumors, there is a balance between hypoxia-induced adaptation mechanisms, including activation of the hypoxia-inducible transcription factors (HIFs) and ingrowth of blood vessels that supply the tumor cells with oxygen and nutrients, counteracting the hypoxic milieu.

## Oxygenation of neuroblastomas

To our knowledge, direct measurements of oxygen tensions in neuroblastoma patient specimens have not been carried out, or at least reported. It is likely, though, that most neuroblastomas are less well oxygenated as compared to non-transformed sympathetic ganglia. In particular, fast-growing high-risk neuroblastomas show frequent areas of necrosis and immunostaining for HIFs reveals local high expression of these factors, often in tumor cells adjacent to necrotic zones (Pietras et al. 2008). Thus, although we do not know how the oxygen tension in neuroblastomas varies from one tumor to another, or whether there is a correlation between low oxygenation and tumor aggressiveness as in other solid tumor forms, available data summarized below suggest that hypoxia also contributes to the malignant phenotype in neuroblastoma.

**Fig. 1:**
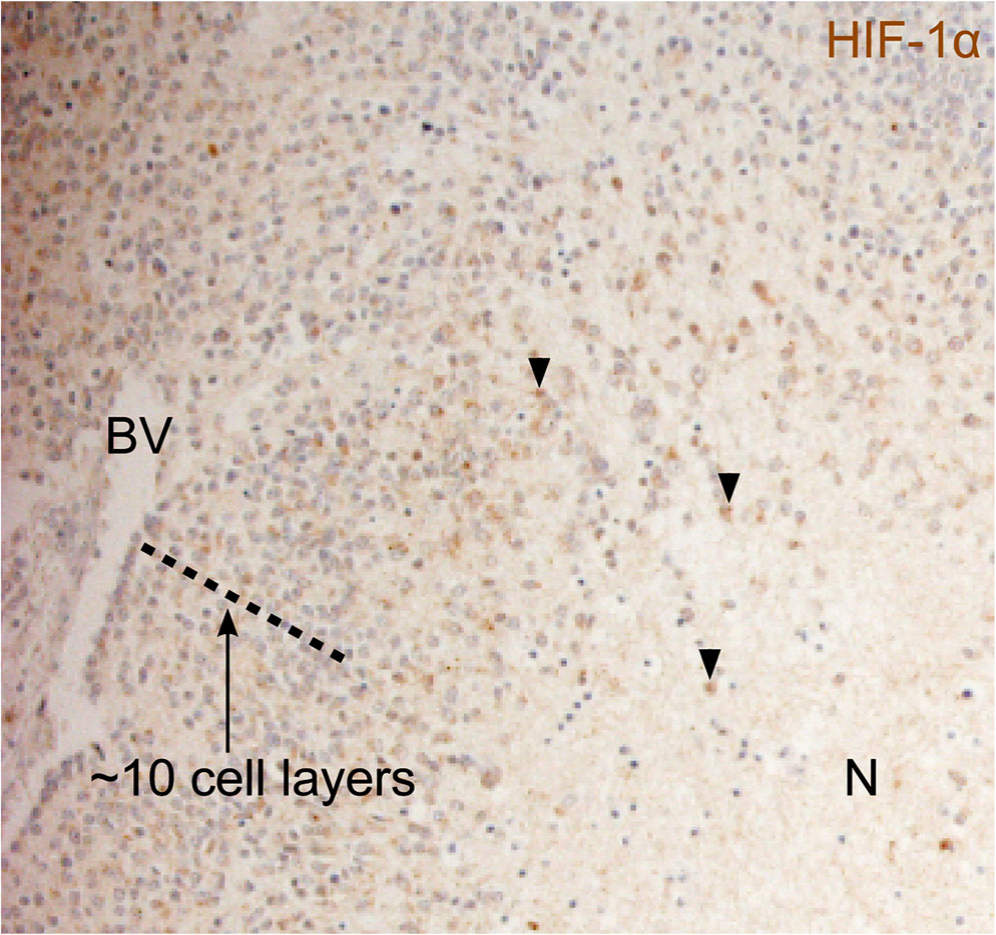
HIF-1α immunohistochemical staining of a necrotic area in a neuroblastoma specimen. Blood vessels can sufficiently supply approximately 10 cell layers with nutrients and oxygen. The hypoxia response machinery becomes activated when oxygen drops below physiological levels, which varies from tissue to tissue but is usually around 3–4% oxygen. *N* necrosis; *BV* blood vessel. *Arrowheads* indicate examples of cells with nuclear HIF-1α expression

One exception to the general conclusion that hypoxia promotes aggressive tumor behavior might be a small subset of neuroblastomas that show a lobular growth pattern and a neuronal-to-neuroendocrine lineage shift in cells close to necrotic areas in the centers of the non-vascularized lobules (Gestblom et al. 1997; Hedborg et al. 1995; Hoehner et al. 1996). This tumor subset is generally not more aggressive than non-lobular neuroblastomas and there is no straightforward correlation between neuroendocrine features and aggressive disease (Fredlund et al. 2008; unpublished data). In these tumor lobules, tumor cells of the outer cell layers, spatially closest to blood oxygen support, display an immature neuronal phenotype that gradually shifts toward a neuroendocrine gene expression pattern and neuroendocrine morphology as the cells are positioned closer to the necrotic zone (Gestblom et al. 1997). The assumption that the neuroendocrine tumor cells, based on their positions in these specimens, are hypoxic is supported by their HIF-1α positivity (Fig. 1).

To investigate the effects of hypoxia on the neuroblastoma phenotype with the intention to unravel whether or not hypoxia promotes neuroendocrine features, we exposed established neuroblastoma cell lines to low oxygen (1% O_2_) for 4 days or more. The hypoxic cells did not shift into a more neuroendocrine state but rather expressed markers of immature, neural crest-like cells (Jögi et al. 2002). These findings, together with similar observations in breast cancer (Helczynska et al. 2008), suggest that, rather than promoting a differentiated phenotype, hypoxia induces immature stem cell-like features, a concept generally accepted today. Nevertheless, the association between hypoxia and an immature neuroblastoma phenotype (Jögi et al. 2002), which in turn associates with poor clinical outcome (Fredlund et al. 2008), was the first indication that hypoxia favors aggressive neuroblastoma features.

## Hypoxia-inducible factors and the hypoxia-response machinery

The core of the active HIF transcription complex consists of one oxygen-sensitive α-subunit (HIF-1α, HIF-2α and HIF-3α) and one oxygen-insensitive β-subunit, also termed ARNT (Semenza 2014). The alpha subunits are encoded by the *HIF1A*, *EPAS1*/*HIF2A* and *HIF3A* genes that generally are transcribed in most cells independent of the cellular oxygenation status (see exception in neuroblastoma cells below) and the transcriptional activities of HIFs are generally considered to be regulated at the protein level. In oxygenated cells, the HIF-α subunits are targeted to degradation by prolyl hydroxylase-dependent hydroxylation of prolyl residues, a process that is oxygen-dependent. The modified α-subunits then become recognized by the von Hippel Lindau (VHL) protein, part of the ubiquitin E3 ligase complex, resulting in ubiquitination and proteasomal degradation of the α-subunits. A second HIF oxygen-dependent silencing mechanism involves the factor inhibiting HIF, which hydroxylates an asparagine residue on the HIF-α subunits and thereby prevents the formation of an active transcription complex between the dimeric HIFs and co-activators p300 and CBP (for a review of the classical oxygen-dependent regulation of HIF activity, see Semenza 2014).

The two most well-studied HIFs, HIF-1 and HIF-2, have slightly different activation kinetics and oxygen concentration ranges at which they are active. While HIF-1, through rapid α-subunit stabilization at oxygen levels around 1–2% (i.e., hypoxia), seems to confer an immediate response to hypoxia, HIF-2 activation generally occurs at prolonged hypoxia (Holmquist-Mengelbier et al. 2006). Interestingly, there seems to be limited deviations in the downstream target genes of HIF-1 and HIF-2 and the main different responses exerted by these two transcription factors might instead be governed by differential tissue expression and time and oxygen intervals at which they are active.

Hypoxia-dependent stabilization of both HIF-1 and HIF-2 seems to peak at around 1% oxygen, indicating that further changes in response to even lower oxygen levels are not HIF-driven. Although the underlying mechanisms are less well established, the unfolded protein response machinery becomes activated at such low oxygen levels, suggesting that basic cell control mechanisms can protect the cell in near-anoxic conditions (Wouters and Koritzinsky 2008).

## Expression of hypoxia-inducible factors in neuroblastomas

**Fig. 2:**
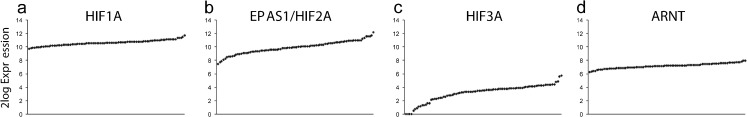
Log2 mRNA expression of *HIF1A*, *EPAS1/HIF2A*, *HIF3A* and *ARNT* in 88 neuroblastomas, data derived from R2: Genomics Analysis and Visualization Platform (http://r2.amc.nl)

Analyses of public gene expression tumor datasets (R2: Genomics Analysis and Visualization Platform (http://r2.amc.nl)) reveal that clinical neuroblastoma specimens express high levels of *HIF1A* and *HIF2A* mRNA, while expression of the less-studied *HIF3A* seems to be detected, if at all, only at extremely low levels (Fig. 2). Consequently, we do not consider *HIF3A* to be a particularly important player in neuroblastoma and will not include the gene or its protein product in our later discussions in this review. All tumors in the R2 dataset examined express robust and somewhat even levels of the HIF binding partner *ARNT* (Fig. 2), an expected pattern considering its oxygen-independent and constitutive expression at protein level.

The activities of HIF-1 and HIF-2 are regulated primarily at the protein level and staining neuroblastomas for expression of HIF-1α and HIF-2α has revealed interesting differences with respect to expression levels in relation to clinical outcome and disease stages. While high HIF-1α levels correlated to low tumor stage and associated with favorable patient prognosis, high HIF-2α expression strongly correlated to unfavorable prognosis and high tumor stage (Holmquist-Mengelbier et al. 2006; Noguera et al. 2009; Zhang et al. 2014). As discussed below, tumors containing cells staining intensely for HIF-2α were generally more aggressive than those lacking these cells, suggesting that HIF-2α marks a neuroblastoma cell type that links to high-risk disease. In a later study, Dungwa et al. reported that HIF-1α levels, either estimated by immunohistochemistry or determined by ELISA, correlate positively with adverse prognostic factors like *MYCN* amplification, 1p deletion and 17q gain (Dungwa et al. 2012). With a cut-off at 10% HIF-1α-positive cells or more, a significant decrease in event-free survival and overall survival was noted but, in a multivariate analysis adjusting for a high-risk group, the significance failed (Dungwa et al. 2012). As discussed in that paper, technical reasons might explain the discrepancy compared with the findings reported by Noguera et al., including cut-off levels for positivity, full-section versus tissue microarray samples analyzed, etc. In addition, one should not underestimate the risk of false positive signals when it comes to anti-HIF-α antibody-based analyses. However, the main conclusions drawn in Dungwa et al., i.e., that HIF-1α expression is elevated in malignant neuroblastomas as compared to ganglioneuromas and that HIF-1α is high in aggressively growing, necrotic tumors, fit the overall assumption that high-risk neuroblastomas are more hypoxic than benign tumors. In a more recent study, Applebaum et al. defined a set of genes with expression levels correlating to adverse neuroblastoma patient outcome and which at the same time were activated by hypoxia. In line with the view that high-risk neuroblastomas are more hypoxic than low-risk tumors, a fraction of the presented set of genes were hypoxia-driven genes that indeed were associated with poor patient prognosis (Applebaum et al. 2016).

## Hypoxia-inducible factors, vascularization and clinical outcome in neuroblastoma

VEGF is a downstream target of both HIF-1 and HIF-2 and the expression levels of either of HIF-1α or HIF-2α correlate positively to VEGF expression (Noguera et al. 2009). However, there is not a perfect match between expression levels and the individual cells that express HIF-1α and HIF-2α, suggesting additional mechanisms than just hypoxia to be responsible for stabilized HIF-α protein in neuroblastoma tissues (see “HIF-2α and the perivascular neuroblastoma niche”). The observation that both HIF-1 and HIF-2 correlate positively to VEGF expression is in accordance with in vitro data showing that HIF-1 as an early and HIF-2 as a late, response to hypoxia, activates VEGF expression. However, these data, together with HIF correlations to clinical outcome and published data for VEGF in neuroblastoma and its relationship to clinical outcome, do not add up to a convincing mechanistic model. In the end, the consensus seems to be that VEGF levels do not reflect a high- or low-stage neuroblastoma geno- or phenotype. Further, published data do not provide a coherent picture regarding the impact of the degree of vascularization on tumor aggressiveness (Canete et al. 2000; Meitar et al. 1996; Noguera et al. 2009). As reported and discussed previously (Noguera et al. 2009), neuroblastoma tissue cells, positive for HIF-1α, are generally not strongly VEGF-positive, which might appear counterintuitive. However, if one considers that HIF-1α is only stabilized in hypoxic cells and takes into account that the presence of VEGF leads to rapid neo-vascularization, oxygenation and degradation of HIF-1α, the apparently contradicting findings may have their explanation. As expected, HIF-1α expression and the presence of blood vessels correlate negatively, supporting a model where HIF-1 is activated by hypoxia via stabilization of HIF-1α, followed by an increase in VEGF transcription, vascularization and in turn degradation of HIF-1α.

## Differential hypoxia-inducible factor expression pattern and activity in neuroblastoma

As touched upon above, HIF-1 and HIF-2 peak in activity at different time points, where HIF-1α is acutely stabilized and responsible for the initial response to deprived oxygen levels. HIF-2 is active mainly during prolonged phases of hypoxia and, in addition, HIF-2α is also stabilized at more physiological oxygen levels (5% O_2_) in cultured cells (Holmquist-Mengelbier et al. 2006). This corresponds well to the findings that clinical neuroblastoma specimens contain rare collections of intensely HIF-2α-positive tumor cells located adjacent to blood vessels (Pietras et al. 2008). Importantly, these tumor cells lack expression of HIF-1α and are thus not hypoxic but instead express several markers associated with an immature neural crest-like phenotype. Considering that HIF-2 can initiate transcription of VEGF at physiological oxygen concentrations in vitro (Holmquist-Mengelbier et al. 2006), it is plausible that the stem cell-like HIF-2α-positive tumor cells are responsible for VEGF expression and neovascularization in the described tumor areas, possibly contributing to their own perivascular niche. If this is true, HIF-2α acts as not only a marker for a subpopulation of neural crest- and stem-cell-like neuroblastoma cells but also works actively to promote an aggressive vascularized phenotype of the tumor.

## HIF-2α and the perivascular neuroblastoma niche

When staining for HIF-2α, with antibodies and under conditions when the antibody specificity is controlled, HIF-2α protein is consistently present in the cytoplasm of neuroblastoma cells. Interestingly, this is noted in particular at physiological oxygen tensions. To exclude unspecific staining as an explanation for cytoplasmic localization and the presence of HIF-2α in non-hypoxic cells, neuroblastoma cells fractionated into cytoplasmic and nuclear fractions were analyzed by western blotting. The data obtained unequivocally demonstrated that HIF-2α can be cytoplasmic, more so at 5% oxygen and that the fraction of cytoplasmic HIF-2α is drastically reduced in hypoxic cells (Holmquist-Mengelbier et al. 2006). These observations are central in order to accept and understand the observations that HIF-2α acts as both a nuclear and a cytoplasmic protein in a subpopulation of vascularized neuroblastoma cells (Pietras et al. 2008). Indeed, this also extends to findings of HIF-2α expression in, e.g,. tumor macrophages (Talks et al. 2000) and during normal development of the sympathetic nervous system ganglia (Mohlin et al. 2013; Tian et al. 1998). The presence of active HIFs under physiological tissue oxygen conditions defines a novel state, a so-called pseudohypoxic state, also allowing for HIF-driven gene expression in oxygenated areas. HIF activity in the presence of oxygen (i.e., pseudoypoxia) can in some cases be dictated by intrinsic signaling pathways (Mohlin et al. 2015). In other cases, normoxic activation of HIFs arises from VHL mutations or inactivation of the prolylhydroxylase dioxygenases by conditions such as iron- or 2-ketoglutarate depletion, or the accumulation of succinate or succinate analogs. According to our view, the pseudohypoxic state may well account for a HIF-driven stem cell phenotype (for further discussion, see Mohlin et al. 2017).

The textbook view is that HIF-2α acts as a crucial subunit of the HIF-2 transcription complex and, thus, exerts its effects via the transcription of downstream target genes. This may still very well be the main function and role of HIF-2α in the cellular response to hypoxia but, recently, a completely different hypoxia-dependent, ARNT-independent, mechanism of HIF-2α action in the cytoplasm has been reported (Uniacke et al. 2012). As a general energy-saving hypoxic response, cells dampen protein synthesis by sequestering the translation initiation factor 4E (eIF4E) from the 5’cap of mRNAs, thus repressing cap-mediated protein synthesis. Still, there is a need for hypoxic cells to synthesize specific proteins taking part in the adaptation process. Uniacke et al. described an oxygen-regulated translation initiation complex, including HIF-2α (although not in complex with, or dependent on, ARNT), the eIF4E homolog eIF4E2 and RNA binding protein RBM4. The HIF-2α–eIF4E2–RBM4 complex can bind to RNA hypoxia response elements present in a large, yet to be defined, set of mRNAs and initiate 5’cap-polysome translation. Since this process takes place in the cytoplasm, this novel HIF-2α-dependent mechanism further provides a rationale for cytoplasmic HIF-2α. As we consistently find cytoplasmic HIF-2α immunoreactivity in stainings and in cytoplasmic cell fractions, including in cells located in pseudohypoxic niches (see, e.g., Holmquist-Mengelbier et al. 2006; Pietras et al. 2008), we postulate that cytoplasmic HIF-2α either serves as a pool to be recruited by ARNT into the nucleus to form an active transcription complex and/or presents its own function in the cytoplasm. One such function would be hypoxia-dependent translational control as described above. Whether the HIF-2α–eIF4E2–RBM4-dependent translation mechanism also functions in physiological conditions where HIF-2α is present is an open question but, if so, this might be a major mechanism, together with HIF-2-dependent transcription, to regulate the phenotype of pseudohypoxic cells and, in the case of neuroblastoma, an immature stem cell-like phenotype.

Few studies have investigated the impact of microRNA-regulated expression of HIF-2α in neuroblastoma but, interestingly, in relation to the 5′-cap-dependent translation described above, Qu et al. identified miR-558 as a crucial regulator of HIF-2α (Qu et al. 2016). Mechanistically, miR-558 binds directly to the 5′-UTR of HIF-2α to facilitate binding between Argonaute 2 (AGO2) and eIF4E, actively promoting HIF-2α translation. Whether microRNA-directed regulation also applies to the HIF-2α–eIF4E2–RBM4-driven translation of HIF-2α itself remains to be investigated but the array of mechanisms through which HIF-2α exerts its effects has definitely broadened in recent years.

## Oxygen-independent activation of hypoxia inducible factors

Although the main regulator of HIF-α stabilization and HIF activation is solely oxygen availability, there are other established routes to induce HIF activity. Examples of normoxic regulation of HIF-2α in neuroblastoma have recently been described to occur via miR-145 (Zhang et al. 2014) and via the deubiquitylase Cezanne and binding of E2F1 (Moniz et al. 2015). While oxygen concentrations uniformly stabilize HIF expression in all adjacent cells, activation by other mechanisms seems to be much more tissue- and time-specific. Growth factors are important regulators of basic cellular functions, such as growth and survival and, indeed, VEGF can be induced by growth factor-mediated HIF-1α expression. Growth factors like insulin-like growth factor (IGF)-I and brain-derived neurotrophic factor (BDNF) were early on described to regulate VEGF expression via elevated HIF-1α protein levels in neuroblastoma cells (Beppu et al. 2005, Nakamura et al. 2006). Inhibitors of either IGF-I or BDNF reduced HIF-1α-mediated VEGF and, interestingly, so did inhibitors of the PI3K/mTOR pathway, suggesting that growth factor-induced stimulation was exerted via PI3K/mTOR signaling (Beppu et al. 2005; Nakamura et al. 2006). Oxygen-independent regulation of HIF-1α has also been extensively studied in cancer forms other than neuroblastoma, where insulin (Treins et al. 2002), epidermal growth factor (Zhong et al. 2000) and VEGF itself (Calvani et al. 2008) can promote HIF-1α expression. It has only recently been demonstrated in neuroblastoma that HIF-2α can also be regulated via growth factor-induced signaling. In contrast to HIF-1α, a large part of this regulation is governed at the transcriptional level, where IGF-II-induced PI3K-mTORC2 signaling promotes *HIF2A* expression at normoxic, near-physiological and hypoxic conditions (Mohlin et al. 2013, 2015). Activating mutations in either of the various growth factors or their receptors, or downstream signaling pathways, which can induce HIF expression, have been detected in various cancer forms. Of interest, HIF-2α itself has also been found to be mutated in cases of paragangliomas (Comino-Mendez et al. 2013; Yang et al. 2013; Zhuang et al. 2012), together suggesting that the hypoxic and pseudo-hypoxic tumor phenotypes are intriguing therapeutic cancer targets.

## HIF-2 as a putative treatment target in neuroblastoma

We postulated that HIF-2 would be an interesting target in neuroblastoma, based on our observations that high HIF-2α associates with an aggressive tumor phenotype and that cells with intense HIF-2α expression are frequently located in perivascular niches and that these tumor cells are stem cell- and mesenchymal-like (Holmquist-Mengelbier et al. 2006; Pietras et al. 2008). One treatment strategy would be to initiate differentiation of the immature HIF-2α-positive cells by inhibiting HIF-2 and then treat these differentiated “bulk”-like tumor cells with conventional therapy. The first part of this treatment regimen—to induce neuronal sympathetic differentiation in stem cell-like neuroblastoma cells with high HIF-2α expression—has been demonstrated in practice (Pietras et al. 2009). Further work with subpopulations of neuroblastoma cells, isolated to express high HIF-2α and present with aggressive tumor cell features, in combination with novel HIF-2 targeting strategies and conventional therapy, may lead us into promising new treatment avenues for this tumor type.

HIF-2 is, however, not only an attractive therapeutic target in neuroblastoma but also in several other tumor forms including glioblastoma, clear cell renal cell carcinoma (ccRCC), paraganglioma and pheochromocytoma (reviewed in Wigerup et al. 2016). Based on structural studies of the HIF-2α–ARNT interaction, a novel inhibitor of specifically HIF-2 transcriptional activity has been presented (Scheuermann et al. 2013). Mechanistically, the small molecule PT2385 acts as a ligand and binds to a cavity present in the PAS-B domain of HIF-2α, prohibiting as well as reversing HIF-2 dimerization, DNA binding and thereby transcriptional activation of downstream target genes (Scheuermann et al. 2013). PT2385 and its analogue PT2399, have been primarily tested in preclinical models of ccRCC, as this tumor form is in great part driven by mutations in VHL, leading to stabilization of, in particular, HIF-2α. Treatment of ccRCC cell lines, with conventional xenograft models as well as patient-derived xenograft models, shows great promise with a large reduction in tumor growth (Chen et al. 2016; Wallace et al. 2016). However, the study by Chen et al. identified a subset of ccRCC tumors that did not respond to PT2399 despite disrupted HIF-2α–ARNT dimerization. These tumors displayed insensitivity to PT2399 when analyzing PT2399-induced gene expression signatures and generally expressed lower levels of HIF-2α than did sensitive tumors (Chen et al. 2016). In addition, xenograft tumors that were initially sensitive to PT2399 treatment with reduced tumor growth developed resistance to PT2399. Resistance could be ascribed to an acquired binding site and second site suppressor mutations, also leading to HIF-2 dimerization in the presence of PT2399 (Chen et al. 2016). These results suggest an immense importance of active HIF-2 in ccRCC and most likely other tumor forms. Also of note, in light of the above, putative important cytoplasmic functions of HIF-2α, PT2385 and PT2399 only work to inhibit nuclear transcriptional activity of HIF-2 and tumors dependent on cytoplasmic, ARNT-independent, functions of HIF-2α would most likely be insensitive to these inhibitors. On a more positive note, an extensively pre-treated ccRCC patient that had developed a sensitive tumor graft showed disease control for more than 11 months when treated with PT2385 (Chen et al. 2016) and PT2385 has also entered a Phase I dose-escalation study in patients with advanced ccRCC (NCT02293980). Further studies will depict whether the inhibition of nuclear and/or cytoplasmic HIF-2α and under which circumstances this would work, is sufficient for tumor regression in patients presenting with ccRCC, neuroblastoma and other tumor forms displaying HIF-2α oncogene addiction.
